# Heterogeneous Information Network-Based Patient Similarity Search

**DOI:** 10.3389/fcell.2021.735687

**Published:** 2021-09-08

**Authors:** Hao-zhe Huang, Xu-dong Lu, Wei Guo, Xin-bo Jiang, Zhong-min Yan, Shi-peng Wang

**Affiliations:** ^1^School of Software, Shandong University, Jinan, China; ^2^Joint SDU-NTU Centre for Artificial Intelligence Research, Shandong University, Jinan, China; ^3^Shandong Provincial Key Laboratory of Software Engineering, Shandong University, Jinan, China

**Keywords:** heterogeneous information network, clinical similarity, electronic health records, patient similarity search, weighted meta path

## Abstract

Patient similarity search is a fundamental and important task in artificial intelligence-assisted medicine service, which is beneficial to medical diagnosis, such as making accurate predictions for similar diseases and recommending personalized treatment plans. Existing patient similarity search methods retrieve medical events associated with patients from Electronic Health Record (EHR) data and map them to vectors. The similarity between patients is expressed by calculating the similarity or dissimilarity between the corresponding vectors of medical events, thereby completing the patient similarity measurement. However, the obtained vectors tend to be high dimensional and sparse, which makes it hard to calculate patient similarity accurately. In addition, most of existing methods cannot capture the time information in the EHR, which is not conducive to analyzing the influence of time factors on patient similarity search. To solve these problems, we propose a patient similarity search method based on a heterogeneous information network. On the one hand, the proposed method uses a heterogeneous information network to connect patients, diseases, and drugs, which solves the problem of vector representation of mixed information related to patients, diseases, and drugs. Meanwhile, our method measures the similarity between patients by calculating the similarity between nodes in the heterogeneous information network. In this way, the challenges caused by high-dimensional and sparse vectors can be addressed. On the other hand, the proposed method solves the problem of inaccurate patient similarity search caused by the lack of use of time information in the patient similarity measurement process by encoding time information into an annotated heterogeneous information network. Experiments show that our method is better than the compared baseline methods.

## 1. Introduction

Patient similarity search has been identified as one of the key techniques in artificial intelligence (AI) medicine service, which is beneficial to medical diagnosis, such as making accurate predictions for similar diseases and recommending personalized treatment plans (Sharafoddini et al., 2017). Generally speaking, patient similarity analysis involves selecting certain clinical records as features of patients in a specific medical environment, then quantitatively analyzing the distance between them. A proper similarity measure should support various downstream applications, such as personalized medicine recommendation (Zhang et al., 2014; Lee et al., 2015), target patient retrieval (Sun et al., 2012), medical diagnoses (Gottlieb et al., 2013), and cohort study (Che et al., 2017).

The wide availability of Electronic Health Records (EHRs) makes it possible to quickly and accurately calculate the similarity between patients. Many similarity learning methods have been proposed (Tsevas and Iakovidis, 2011; Wang et al., 2012b; Barkhordari and Niamanesh, 2015; Wang and Sun, 2015; Sha et al., 2016; Zhan et al., 2016; Sharafoddini et al., 2017; Huai et al., 2018; Suo et al., 2018) on healthcare datasets. Existing methods have successfully derived the similarity measure from EHRs data through mapping the medical events into vector spaces. However, EHRs contain a variety of data (diagnostics, drugs, etc.) and a large number of medical events, which usually results in high-dimensional embedding vectors.

Heterogeneous information network (HIN) contains rich structure and semantic information, and it can effectively solve the problem caused by the high-dimensional and sparse embedding vectors. For calculating the similarity of patients, the diseases and drugs used by patients provide essential information. The patient's disease is critical to the doctor's clinical decision. At the same time, the patient's disease is basically determined by the patient's clinical symptoms and clinical indicators. It can be said that the disease is a comprehensive reflection of clinical indicators. The medicine is the solution made by the doctor to the patient's disease and symptoms, and is the final manifestation of the doctor's clinical decision. Therefore, it is easy to think that patients, diseases, and drugs can be connected to form HIN.

However, there are many duplicate diseases and drugs in the EHRs, meaning that if we were to use classic HIN modeling techniques with the above schema, we would lose the correlation information between patients and drugs. Considering this problem, we propose a kind of HIN with annotation: that is, in links connecting diseases and drugs, we add an annotation of patient information to enrich the original network with the information between patients and drugs. We call it annotated HIN. On the annotated HIN, we propose a novel node similarity measure S-PathSim to calculate patient similarity. As a node similarity measure, S-PathSim enjoys some good properties, like symmetric and self-maximum.

On the other hand, temporal information is crucial to understand the dynamics of medical expressions. To leverage the essential temporal information for patient similarity evaluation, we propose to use N-disease to encode temporal information into annotated HINs. N-disease is inspired by the N-grams model in natural language processing. Its basic idea is to arrange the patients' diseases into time series according to the time they are developed, sequentially collect the N-grams from the disease sequences, and then replace the disease object with the disease N-grams in the annotated HIN. The collected N-grams from the disease time series are called N-diseases.

Finally, two patient similarity search methods, MBH (method based on annotated HIN) and MBHT (method based on annotated HIN and temporal information), were defined according to S-PathSim and N-disease.

The remainder of this paper is structured as follows. The second section reviews the related research work on the topic of patient similarity analysis and heterogeneous information network, while the third section provides some preliminaries on HIN and shows the limitation of HIN to the calculation of patient similarity. In the fourth section, we introduced our method in detail. The experimental results and comparative analysis are shown in section five. Finally, the last section summarizes this paper and discusses some possible avenues for future research.

## 2. Related Work

In this section, we review some related works on evaluating patient similarity and heterogeneous information network.

Studying patient similarity has practical significance in many applications (Lee et al., 2015; Li et al., 2015). Ng et al. provided personalized predictive healthcare model by matching clinical similar patients with a locally supervised metric learning measure (Ng et al., 2015). An integrated method for personalized modeling (IMPM) was proposed to provide personalized treatment and personalized drug design (Kasabov and Hu, 2010). The data-driven clinical decision support system was combined with patient similarity (Xia et al., 2019).

At present, there are many studies to calculate the similarity of patients. Zhang et al. combined patient similarity and drug similarity analysis and proposed a heterogeneous label propagation method to identify which drug is likely to be effective for a given patient (Zhang et al., 2014). Chan et al. proposed a patient similarity algorithm named SimSvm that uses support vector machine to weight the similarity measures (Chan et al., 2010). Wang et al. proposed a patient similarity based disease prognosis strategy named SimProX (Wang et al., 2012a). This model used a local spline regression based method to embed these patient events into an intrinsic space, and then measure the patient similarity by the Euclidean distance in an embedded space. However, these methods do not leverage temporal information to evaluate patient similarities, which prevents them from delivering.

Cheng et al. (2016) took temporal information into consideration and proposed an adjustable temporal fusion scheme using CNN-extracted features. This method is a supervised model, but the label data are not easy to obtain, which limits its use, and the method lacks interpretability. Zhu et al. proposed the method to solve the problem of high-dimensional vectors and time series (Zhu et al., 2016). They embed medical events from HER into fixed-length vectors, but fixed-length vectors are difficult to obtain complete medical event information.

As mentioned above, the current method of measuring patient similarity is limited, and a better method is needed to calculate patient similarity.

Since, Sun et al. proposed the concept of HIN (Sun and Han, 2010), and the meta path concept subsequently (Sun and Han, 2011), HIN analysis becomes a hot topic rapidly in the fields of data mining, database, and information retrieval. He et al. incorporated temporal information for similarity search in HINs by assigning different weights to the paths built at different time (He et al., 2014). But this method is not suitable for the annotated HIN proposed in this paper. In order to evaluate the relevance of different-typed objects, Shi et al. (2014) proposed HeteSim to measure the relevance of any object pairs under arbitrary meta paths. As an adaption of HeteSim, LSH-HeteSim (Li et al., 2014) is proposed to mine the drug–target interaction in heterogeneous biological networks where drugs and targets are connected with complicated semantic paths. In order to overcome the shortcoming of HeteSim in high computation and memory demand, Meng et al. (2014) proposed the AvgSim measure that evaluates similarity score through two random walk processes along the given meta path and the reversed meta path, respectively. In order to overcome the problem that the meta path can only express simple information, Cheng et al. (2017) proposed meta structure to measure the similarity between the objects. Until today, HINs have been widely used in other fields (Wang, 2019; Wang et al., 2020; Zhang et al., 2020).

HIN rarely results in high-dimensional vectors, and most similarity calculation methods based on HIN have good interpretability. But it cannot be perfectly applied to patient similarity calculation, so in this paper, we propose an improved method, annotated HIN, which can be well-applied to calculate the similarity of patients.

## 3. Preliminaries

In this section, as preliminaries, we will detail the HIN and its limitation in measures patient similarity.

### 3.1. HIN

An information network is defined as a directed graph *G* = (*V, E*) with an object type mapping function ψ: *V* → *A* and a link type mapping function φ: *E* → *R*, in which each object *v* ∈ *V* belongs to a particular object type ψ (*v*) ∈ *A* while each link *e* ∈ *E* belongs to a particular relation φ (*e*) ∈ *R*.

Different from the traditional network definition, we explicitly distinguish the object types and relationship types in these networks. When the types of objects |*A*| > 1 or the types of relations |*R*| > 1, the network is referred to as a heterogeneous information network; otherwise, it is a homogeneous information network.

### 3.2. Limitation of HIN

**Figure 1:**
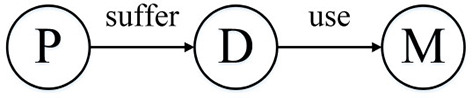
Network schema of the patient heterogeneous information network.

HIN can link patients, diseases, and drugs. As shown in Figure 1, we can get the network schema of the patient HIN. P, D, and M represent patient, disease, and medicine, respectively.

There may be many kinds of drugs to treat one disease, and one drug can also cure many diseases, which leads to some incorrect information in the traditional heterogeneous information network when connecting patients, diseases, and drugs. We use a specific example below to illustrate this problem.

**Figure 2:**
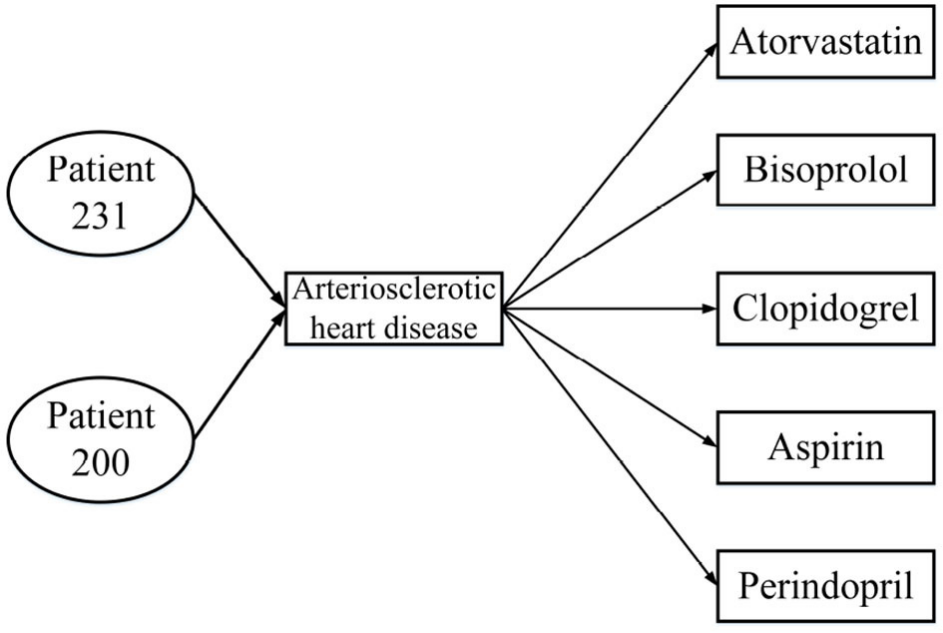
Heterogeneous information network from Table 1.

Table 1 presents three inpatient records for two patients, all of which were diagnosed with the same disease; patient 231 was hospitalized twice. From the data in Table 1, the HIN in Figure 2 is obtained. However, the HIN shown in Figure 2 has two problems. First of all, we need to know that patient 231 has been hospitalized twice, but this information cannot be obtained through Figure 2. Second, patient 231 does not use perindopril in treatment, but the information we get from the heterogeneous information network is that there is a relation between patient 231 and perindopril, which leads to the incorporation of misleading information. Therefore, traditional HIN-based measurement methods are not suitable for our problem.

**Table 1:** Example of case information.

**Hospital ID**	**Patient ID**	**Disease**	**Medicine**
564435	231	Arteriosclerotic heart disease	Atorvastatin, Bisoprolol, Clopidogrel
561657	200	Arteriosclerotic heart disease	Aspirin, Atorvastatin, Perindopril
564677	231	Arteriosclerotic heart disease	Atorvastatin, Clopidogrel

## 4. The Proposed Method

### 4.1. Annotated HIN

As mentioned in section 3, HIN is not suitable for our problem. In order to measure patient similarity, we propose a new graph model-annotated HIN.

**Definition 1**. ***Annotated Heterogeneous Information Network*.** Annotated HIN is a special heterogeneous information network *G* = (*V, E, C*). In the annotated HIN, there is a set of one or more link types annotated by < *key, value* > pairs. For each < *key, value* > pair, key corresponds to a specific type of object ψ (*key*) ∈ *V*, while value is used to record the number of links.

As above mentioned, we regard the set of < *key, value* > pairs as the annotations of a heterogeneous information network, represented by *C*. The number of key-value pairs in the set is referred to the length of the annotation, which is represented by *L*. Annotations can be added to one or more link types of the classic heterogeneous information network. These annotations can be used to record the source and number of connections and can thus represent more information.

**Figure 3:**
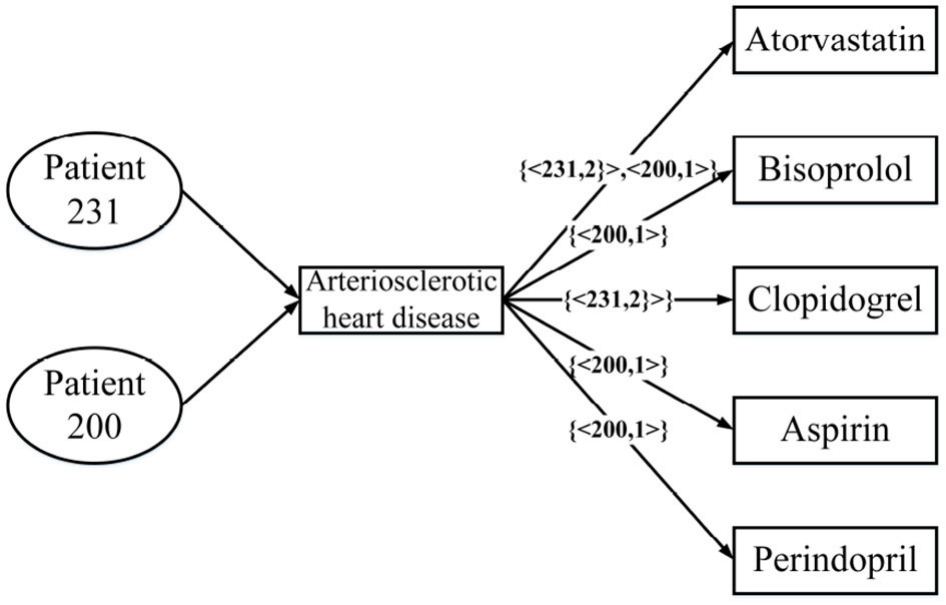
An annotated HIN from Table 1.

Figure 3 is a real example diagram of an annotated HIN, and we named it patient-annotated HIN. It can be seen that the connection with “Clopidogrel” has annotation *C*_*Clopidogrel*_ = {< 231, 2 >}, and that the annotation length is *L* = 1. Combined with the annotated heterogeneous information network, we can interpret it as follows: Patient 231 was diagnosed with atherosclerotic heart disease in both hospitalizations, and clopidogrel was used in both treatments. Moreover, there is no corresponding record of patient 200 in the note, so it can be concluded that clopidogrel was not used in the treatment of patient 200. In this way, the two problems described in the previous section are solved.

**Figure 4:**
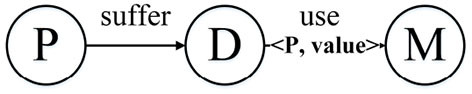
Schema of patient-annotated HIN.

For a given annotated HIN, in order to help readers better understand the object type, link type, and annotation type in the network, we provide its meta-description.

**Definition 2**. ***AHIN Network Schema*. **The network schema of AHIN is recorded as *SG* = (*A, R, I*). This is a meta template of AHIN *G* = (*V, E, C*). It has object type mapping ψ (*v*) ∈ *A*, relation type mapping φ (*e*) ∈ *R*, and annotation type mapping θ:*C* → *I*. It is defined on object type set *A*, relation type set *R*, and annotation type set *I*.

### 4.2. Weighted Meta Path and S-PathSim

The weighted meta path, designed to capture complex relationship between two annotated HIN objects, is based on network expansion structure. And the network expansion structure is defined as follows.

**Definition 3**. ***Network Expansion Structure*. **Network expansion structure *S* is a set of directed weighted graphs, which is defined on an annotated HIN schema *SG* = (*A, R, I*). It expands the annotated heterogeneous information network into an easy-to-process format. Formally, *S* = (*D*_1_, *D*_2_, …, *D*_*n*_), where *D*_*n*_ = (*V*_*n*_, *E*_*n*_) is a directed weighted graph with *D*_*n*_, *V*_*n*_ being the set of nodes and edges, respectively. For any edge *e* ∈ *E*_*n*_, a weight *w*(*e*) is associated, with the default value 1.

**Figure 5:**
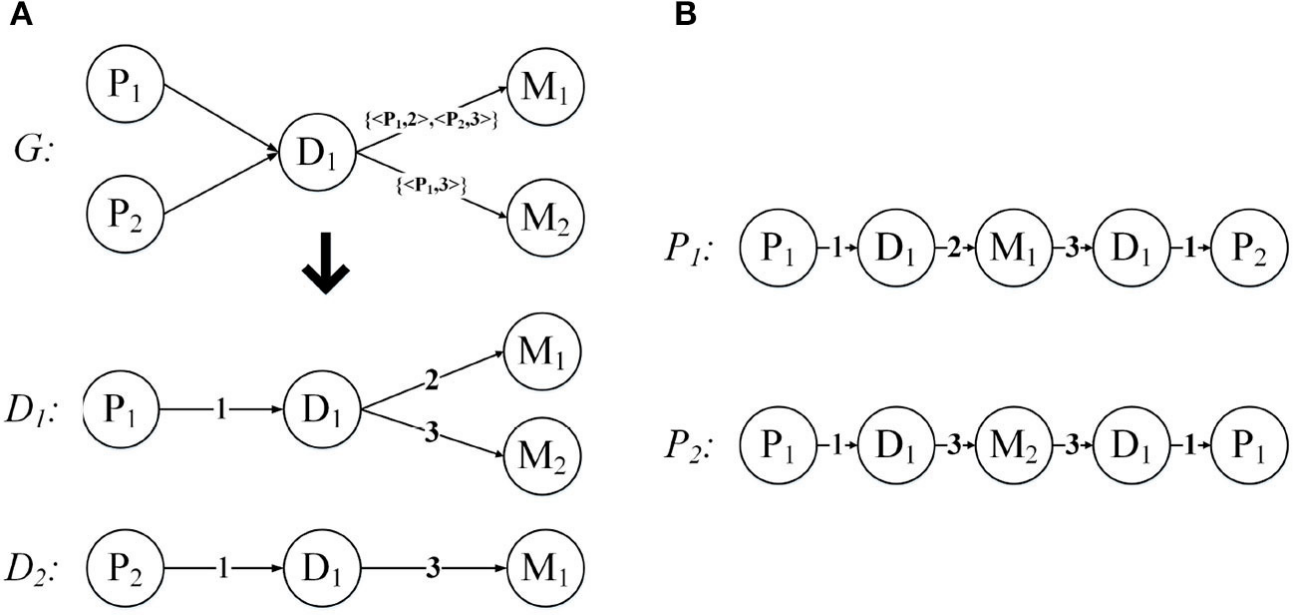
Network expansion structure and weighted meta path. **(A)** Network expansion structure. **(B)** Weighted meta path.

Below we use an example to introduce the expansion of the network structure. Figure 5A demonstrates the expansion from a given annotated heterogeneous information network into the network expansion structure. There are annotations {< *P*_1_, 2 >, < *P*_2_, 3 >}, and {< *P*_1_, 3 >} in graph *G*. The key-value pairs < *P*_1_, 2 > and < *P*_1_, 3 > correspond to the entity *P*_1_, so we can get the graph *D*_1_, and the corresponding edge weights are 2, 3, respectively. And the key-value pair < *P*_2_, 3 > corresponds to the entity *P*_2_, so we get the graph *D*_2_, and the corresponding edge weight is 3. For the other edges, our default weight is 1.

After introducing the network expansion structure, we propose the concept of weighted meta path.

**Definition 4**. ***Weighted Meta Path*. **Weighted meta path *P* is a path defined on the network schema *SG* = (*A, R, I*), and based on network expansion structure *S* = (*D*_1_, *D*_2_, …, *D*_*n*_). Weighted meta path is denoted in the form of $A_{1}\overset{R_{1},w\left(e_{1}\right)}{\to }A_{2}\overset{R_{2},w\left(e_{2}\right)}{\to }\ldots \overset{R_{l},w\left(e_{l}\right)}{\to }A_{l+1}$, which defines a composite relation between object *A*_1_ and *A*_*l*+1_, where *R*_*l*_ represents the relationship between *A*_1_ and *A*_*l*+1_, and *w*(*e*_*l*_) represents the weight of the relationship.

Just like the meta path, if the relationship of the weighted meta path *P* is symmetric, then we say that it is symmetric. For a specified weighted meta path, it has a specified template. If there is no multiple relationship between the same object types, we can use the type name to represent the template of the weighted meta path: *P* = (*A*_1_*A*_2_…*A*_*l*+1_). As shown in Figure 5B, *P*_1_ and *P*_2_ have the same template *PDMDP*. *P*_1_ and *P*_2_ are symmetric weighted meta paths.

When $A_{l+1}=A_{1}^{\prime }$, the weighted meta paths *P* = (*A*_1_*A*_2_ … *A*_*l*+1_) and $P^{\prime }=\left(A_{1}^{\prime }A_{2}^{\prime }\ldots A_{l+1}^{\prime }\right)$ are concatenable, so that a new weighted meta path $\left(A_{1}A_{2}\ldots A_{l+1}A_{2}^{\prime }\ldots A_{l+1}^{\prime }\right)$ is obtained.

**Figure 6:**
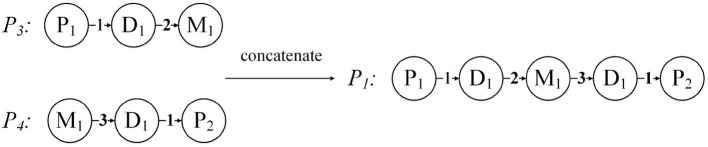
Concatenation of weighted meta path.

For each weighted meta path *P*, there is a score *S*(*P*), and *S*(*P*) is the product of the weights of the relationships in *P*. For example, the weighted meta path *P*_1_, *S*(*P*_1_) = 1 * 2 * 3 * 1 = 6. In fact, *S*(*P*) represents the weight of the relationship between the first and last objects in the weighted meta path *P*, and can also be understood as the number of connection paths between the two objects. As shown in Figure 6, the weighted meta path *P*_3_, *W*({_*D*_1_, *M*_1_}*P*3_) = 2, represents that patient *P*_1_ has used the drug *M*_1_ twice because of disease *D*_1_. Therefore, *S*(*P*_3_) = *W*({_*P*_1_, *D*_1_}*P*_3__) * *W*({_*D*_1_, *M*_1_}*P*_3__) = 2 can also be obtained, then the number of connection paths between *P*_1_ and *M*_1_ is 2. In the same way, *S*(*P*_4_) = *W*({_*M*_1_, *D*_1_}*P*_4__) * *W*({_*D*_1_, *P*_2_}*P*_4__) = 3, then the number of connection paths between patient *P*_2_ and drug *M*_1_ is 3. *P*_1_ can be obtained by concatenating *P*_3_ and *P*_4_, then we can get that the number of connection paths between patient *P*_1_ and patient *P*_2_ is *S*(*P*_1_) = *S*(*P*_3_) * *S*(*P*_4_) = 6.

Based on the annotated HIN and weighted meta path, we propose a new measure, named S-PathSim.

**Definition 5**. ***S-PathSim*. **Given a symmetric weighted meta path, S-PathSim between two objects of the same type x and y is:

(1) $$\begin{array}{l} s\left(x,y\right)=\frac{2\times S_{sum}\left(P_{x\to y}\right)}{S_{sum}\left(P_{x\to x}\right)+S_{sum}\left(P_{y\to y}\right)}, \end{array}$$

where *S*_*sum*_(*P*_*x*→*y*_) is the sum of score of the weighted meta path between *x* and *y*, *S*_*sum*_(*P*_*x*→*x*_) is that between *x* and *x*, and *S*_*sum*_(*P*_*y*→*y*_) is that between *y* and *y*. If there are two weighted meta-paths *P*_*a*_ and *P*_*b*_ between *x* and *y*, and *S*(*P*_*a*_) = 4, *S*(*P*_*b*_) = 3, then *S*_*sum*_(*P*_*x*→*y*_) = *S*(*P*_*a*_) + *S*(*P*_*b*_) = 7.

Take the patients in Table 1 as an example, and patient 231 has two admissions. During his first hospitalization, he developed arteriosclerotic heart disease and had some medicine including atorvastatin, bisoprolol, and clopidogrel. Patient 200 also developed arteriosclerotic heart disease and he had the medicine aspirin, atorvastatin, and perindopril. According to these information, we can get an heterogeneous information network G as shown in Figure 5A. According to Definition 5, we can get *S*_*sum*(*patient*231 → *patient*200) = 6, *S*_*sum*_(*patient*231 → *patient*231) = 22, *S*_*sum*_(*patient*200 → *patient*200) = 9, therefore *s*(*patient*231, *patient*200) = 6/11.

As mentioned before, *S*(*P*) can be understood as the number of connecting paths of the first and last two objects in the weighted meta path *P*. If there are more connection paths between two objects, then we can consider them to have a higher similarity. However, the result obtained by using the number of paths as the judgment condition will be biased toward high-visibility objects. Therefore, we use the number of connection paths from two objects to their own as a balance factor. This idea has been applied to PathSim, and we extend it to the annotated HIN here, and propose S-PathSim.

Properties of S-PathSim:

- (1) Symmetric: *s*(*x, y*) = *s*(*y, x*). Considering the semantics of *S*_*sum*_(*P*_*x*→*y*_), it is easy to understand *S*_*sum*_(*P*_*x*→*y*_) = *S*_*sum*_(*P*_*y*→*x*_), so *s*(*x, y*) = *s*(*y, x*).
- (2) Self-maximum: *s*(*x, y*) ∈ [0, 1], and *s*(*x, x*) = 1. The weighted meta path template *mn* and *nm* can be concatenated into a new weighted meta path *mnm*.*mnm*_*i*_ is the *i*th path of the weighted meta-path template *mnm*, as mentioned before, *S*(*mnm*_*i*_) = *S*(*mn*_*i*_) * *S*(*nm*_*i*_). Assuming that *mn* is the weighted meta path template, the *k*th weighted element path is expressed as *a*_*k*_, and *nm* is the weighted meta path template, and the *k*th weighted element path is expressed as *b*_*k*_, then $S_{sum}\left(P_{x\to y}\right)=\overset{p}{\underset{k=1}{\sum }}S\left(a_{k}\right)*S\left(b_{k}\right)$; the same can be obtained as $S_{sum}\left(P_{x\to x}\right)=\overset{q}{\underset{k=1}{\sum }}S{\left(a_{k}\right)}^{2}$, $S_{sum}\left(P_{y\to y}\right)=\overset{o}{\underset{k=1}{\sum }}S{\left(b_{k}\right)}^{2}$. There must be *p* ≤ *q, p* ≤ *o*. Then $2\overset{p}{\underset{k=1}{\sum }}S\left(a_{k}\right)*S\left(b_{k}\right)\leq \overset{p}{\underset{k=1}{\sum }}S{\left(a_{k}\right)}^{2}+\overset{p}{\underset{k=1}{\sum }}S{\left(b_{k}\right)}^{2}\leq \overset{q}{\underset{k=1}{\sum }}S{\left(a_{k}\right)}^{2}+S{\left(b_{k}\right)}^{2}$, so *S*(*x, y*) ≤ 1. And it is easy to understand that *s*(*x, y*) ≥ 0, so *s*(*x, y*) ∈ [0, 1], *s*(*x, x*) = 1. In the above formula, *p* represents the number of weighted meta path between *x* and *y*, *q* represents the number of weighted meta path between *x* and *x*, and *o* represents the number of weighted meta path between *y* and *y*.

### 4.3. Temporal Information Encoding

Temporal information is critical to understanding the patients' dynamics. However, the AHIN described previously cannot capture the temporal information, so for the problem to be solved in this article, we propose an N-disease method to embed temporal information into the AHIN.

**Figure 7:**
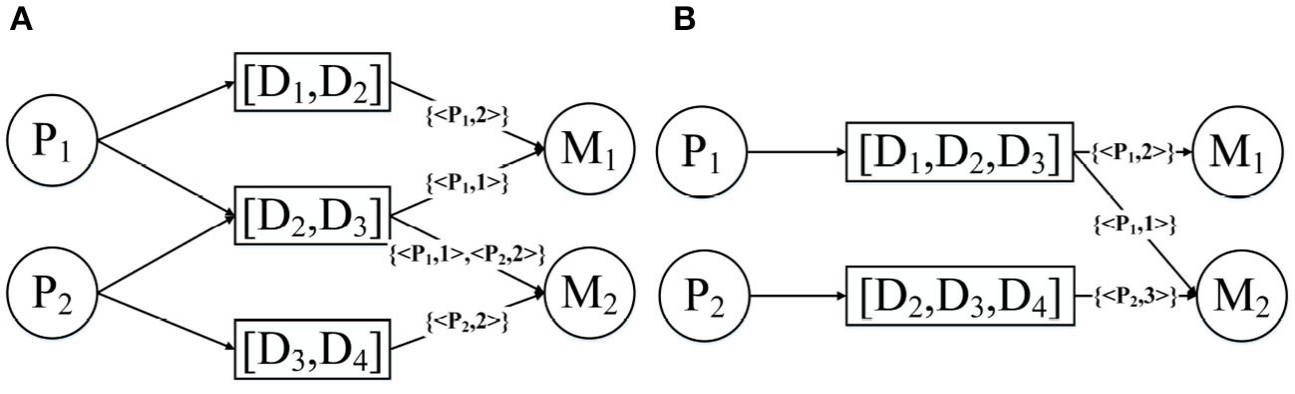
Annotated HIN after **(A)** 2-disease and **(B)** 3-disease operations.

N-disease is inspired by the natural language processing model N-grams. Its basic idea is to arrange the patients' diseases set into time series according to the time when they were developed, sequentially collect the N-grams from the disease sequences, and then replace the disease object with the disease N-grams in the annotated HIN. Assuming that P_1_ has the diseases [*D*_1_, *D*_2_, *D*_3_] and P_2_ has the disease [*D*_2_, *D*_3_, *D*_4_], then the results obtained after the 2-disease operation and the 3-disease operation are shown in Figure 7. In fact, the patient annotation HIN given in Figure 4 is essentially the patient annotation HIN after 1-disease operation.

It should be noted that as *N* becomes larger and larger, the accuracy of the patient's annotation of diseases and drug connections in the HIN will gradually decrease. As shown in Figure 7A, the node [*D*_1_, *D*_2_] is connected to the drug; then you do not know whether this drug is used to treat disease *D*_1_ or disease *D*_2_. Fortunately, we can trade off the accuracy and temporal information by changing *N*.

### 4.4. MBH and MBHT

Retrieving top-k similar patients of specified patients has practical significance. It allows doctors to analyze similar patients to provide better treatment options. Previously, we have introduced the annotated HIN–based measurement method S-PathSim and temporal information embedding method N-disease. In this section, we define two patient similarity search methods, MBH and MBHT, according to the definition introduced earlier.

MBH is a method based on annotated HIN. In detail, first, annotated HIN is constructed using the patient's medical record information. After specifying a patient, S-PathSim is used to calculate the patient similarity and return the top-k similar patient.

MBHT is a method based on annotating HIN and temporal information. The difference between MBHT and MBH is that MBHT needs to construct the annotated HIN processed by the N-disease based on patient's medical record information, and embed the temporal information into the annotated HIN, then use S-PathSim to calculate the patient similarity and return the top-k similar patient.

It is easy to understand that MBHT is the combination of N-disease and MBH. When *N* = 1, MBHT is MBH. MBHT uses the temporal information in the patient's medical records, but it also loses some accuracy, and we need to make a trade-off between timing and accuracy.

## 5. Simulation Experiments and Results Analysis

### 5.1. Data Description

We perform experiments on a real dataset, which primarily includes information about the medical treatments and drug details of each person. Each person has multiple records (*n* > 2). Moreover, each record contains a diagnosis (i.e., ICD10) and information about multiple drugs. To improve the experiment quality, we randomly divided the data into four sub-datasets. Table 2 shows the description of the divided datasets. In addition, we did not perform any other desensitization treatment (such as removing diseases with less than five patients), so our experiment is performed on a real-world dataset without any unjustifiable data manipulations.

**Table 2:** Description of datasets.

**Sub-dataset**	**Dataset A**	**Dataset B**	**Dataset C**	**Dataset D**	**Total**
Number of patient	13,461	13,461	13,461	13,460	53,853
Disease types	946	953	946	943	1,928
Drug types	1,400	1,412	1,403	1,390	2,217
Number of diagnoses per capita	6.653	6.540	6.647	6.456	6.620

### 5.2. Experimental Settings

In application, comparative analysis is often performed by retrieving top-k similar patients of designated patients to support clinical decision making. In the experiment, we also evaluate the model by retrieving the top-k similar patients of the specified patients. We set *k* = 10. We used two metrics for quantitative evaluation.

*nDCG* (normalized Discounted Cumulative Gain, with the value between 0 and 1, the higher the better) Zhang et al. (2020) is an indicator used to measure the quality of the ranking. The main idea is that the products that the user likes are supposed to be ranked in front of the recommendation list rather than in the back so as to significantly increase the user experience. It is obtained by *DCG* (Discounted Cumulative Gain) normalization, where *rel* is a sorted list, *i* is the position number of the current result, and *IDCG* is the largest *DCG* in the ideal state.

(2) $$\begin{array}{l} DCG=\overset{p}{\underset{i}{\sum }}\frac{rel_{i}}{log_{2}i+1} \end{array}$$

(3) $$\begin{array}{l} nDCG=\frac{DCG}{IDCG} \end{array}$$

The *HL* (half-life utility) (Sarwar et al., 2001) index is proposed under the assumption that the probability that the user browses the product and the specific ranking value of the product in the recommendation list decrease exponentially. It measures the practicality of the recommendation system for a user. It is the difference between the user's actual rating and the model rating. So *HL* can also be used to evaluate top-k search results.

(4) $$\begin{array}{l} HL_{u}=\underset{\alpha }{\sum }\frac{max\left(r_{ua}-d,0\right)}{2^{\left(l_{ua}-1\right)/\left(h-1\right)}} \end{array}$$

Among them, *r*_*ua*_ represents the true similarity of patient *u* and patient *a*, *d* is the default score, in the experiment we set *d* to the average similarity, and *l*_*ua*_ is the ranking of patient *a* in the recommended list of patient *u*. *h* is the half-life of the system, that is, there is a 50% probability that the user will browse the recommended list position, we set *h* = 3.

In order to verify the effectiveness of the proposed MBH based on S-PathSim, we set up a comparison experiment between MBH and the similarity search method based on PathSim. In addition, in order to explore the effect of N-disease on the results, *N* was set to 1, 2, 3, 4, respectively, and count the results of MBHT for comparative analysis. Finally, we explored the effect of N-disease on algorithm efficiency. The experimental environment is as follows: INTELCorei5 CPU, 2.80 GHz; 4G memory.

### 5.3. Comparison of Patient Similarity Search Method

This article proposes annotated HIN and S-PathSim, and defines MBH, a patient similarity search method based on the annotated HIN and S-PathSim. PathSim is an excellent object similarity measurement method based on HIN. PathSim can be used to retrieve the similarity of patients. Here, we compare MBH with PathSim-based methods to verify the effectiveness of MBH:

1. MBH: Map the patient information to the annotated HIN, the schema is shown in Figure 4, through the weighted meta path as shown in Figure 5B; the S-PathSim is used to measure the similarity of patients, and get the top-k similar patients of the specified patients.
2. Baseline: Map patient information into HIN. The schema is shown in Figure 1. The meta path used is (*PDMDP*). PathSim is used to calculate the patient similarity, and the top-k search result of the specified patient is obtained.

It is worth mentioning that the above steps are run simultaneously in 4 sets of datasets, effectively avoiding accidental.

**Figure 8:**
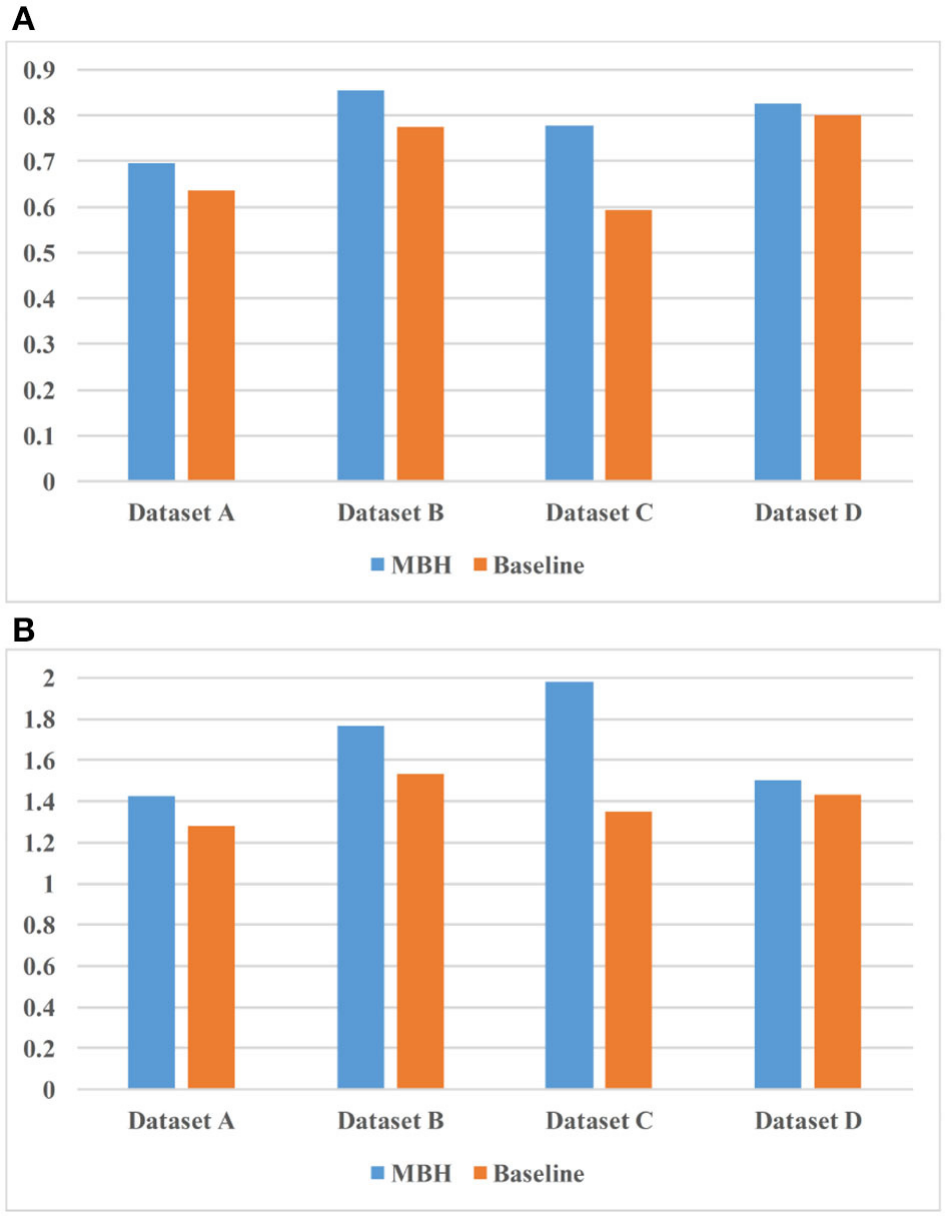
Comparison result of MBH (method based on annotated HIN) and baseline. **(A)** uses nDCG as the evaluation criterion and **(B)** uses HL as the evaluation criterion.

Figure 8 shows the experimental results of the two models on 4 sets of datasets. Figure 8A uses *nDCG* as the evaluation criterion, and it can be observed that MBH is superior to baseline on four datasets. Figure 8B uses *HL* as the evaluation criterion, which proves that MBH has better practicability than baseline.

### 5.4. The Impact of N-Disease

We propose N-disease to embed temporal information into annotated HIN, and the difference between MBH and MBHT is whether N-disease is used or not. In this section, we explore the comparison results of MBH and MBHT, and the effect of N-disease on MBHT. We set *N* to 1, 2, 3, and 4, respectively. When *N* = 1, the annotated HIN does not contain temporal information, and MBHT is MBH. When *N* = 4, annotated HIN contains the largest amount of temporal information. However, after a threshold, with the increase of *N*, the annotated HIN captures increasingly more temporal information while its patient similarity search performance decreases steadily. We should carefully choose the threshold for *N* to obtain the best results.

The experimental results are shown in Table 3. In datasets A, C, and D, MBHT has the best results when *N* = 2; in dataset B, MBHT achieved the best results when *N* = 3. Among the average values of the 4 datasets, *N* = 2 makes MBHT achieve the best results. In general, *N* = 2 can achieve the best results of MBHT, and *N* = 2 can balance the time-consuming and accuracy of annotated HIN. At the same time, the experimental results also show that MBHT is better than MBH.

**Table 3:** The effect of N-disease measured by *nDCG* (normalized Discounted Cumulative Gain).

**Dataset**	***N* = 1**	***N* = 2**	***N* = 3**	***N* = 4**
Dataset A	0.696	**0.704**	0.695	0.694
Dataset B	0.855	**0.859**	0.807	0.761
Dataset C	0.778	0.767	**0.800**	0.791
Dataset D	0.825	**0.837**	0.799	0.787
Mean	0.788	**0.791**	0.775	0.758

*Note: The bold values are the best results*.

In the following, we explore the effect of N-disease on MBHT efficiency. We assume that when N-disease method is not used (i.e., *N* = 1), the running time of the program is unit 1. The experimental results are as follows.

It can be seen from Table 4 that the efficiency of the algorithm is improved by using N-disease; especially when *N* = 2, the algorithm has the highest efficiency. The use of N-disease changes the number of annotated HIN nodes and the relationship between the nodes, which in turn changes the efficiency of the algorithm. Since N-disease will affect the efficiency of MBHT, this paper gives an explanation from a practical point of view.

**Table 4:** The effect of N-disease on efficiency.

**Dataset**	***N* = 1**	***N* = 2**	***N* = 3**	***N* = 4**
Dataset A	1	0.918	0.978	0.972
Dataset B	1	0.828	0.902	0.893
Dataset C	1	0.767	0.957	0.893
Dataset D	1	0.893	0.929	0.954
Mean	1	0.883	0.941	0.928

When the program is implemented, we divide MBHT into two steps. The first step is data statistics, and the second step is S-PathSim calculation. The use of MBHT has more data statistics steps than the use of MBH alone, but we know from practice that the time consumed by the data statistics step is quite small and can even be ignored. When we calculate S-PathSim, we use a lot of multiplication, which takes most of the total running time. We found that when *N* = 2, the number of multiplication operations is significantly smaller than when MBH is used alone. This explains why the running time of the program when *N* = 2 is shorter than that when using MBH alone.

In short, we conclude that when *N* =2, annotated HIN achieves a balance between time consuming and accuracy, and can effectively improve the efficiency of the algorithm.

## 6. Conclusion

In this paper, a new method of patient similarity calculation is proposed that uses the disease and drug data of patients, and further uses the annotated HIN proposed in this paper to create a model. The heterogeneous network adds the annotation of patient information to the connecting links between diseases and drugs, which solves the problem of the classic HIN in losing the information regarding these associations. At the same time, based on the annotated HIN, we propose S-PathSim to measure patient similarity. Furthermore, N-disease is proposed to encode temporal information into the annotated HIN. Our measurement does not rely on high-dimensional and sparse vectors, and effectively captures the patient's medical events and the temporal information in EHRs. Finally, based on S-PathSim and N-disease, two patient similarity search methods, MBH and MBHT, are proposed. The experimental results show that the method proposed in this paper is superior to competitive baseline method.

## Data Availability Statement

The original contributions presented in the study are included in the article/supplementary material, further inquiries can be directed to the corresponding author/s.

## Author Contributions

H-zH designed and conducted the experiments and finished this paper writing. X-dL determined the technical route and methods. WG devised the assessment method for the methods. X-bJ did some research on the related work. Z-mY discussed the methods and ideas. S-pW discussed the methods and ideas and submitted the paper. All authors contributed to the article and approved the submitted version.

## Funding

This work was supported by the National Key Research and Development Project of China (No. 2019YFB1705904), National Nature Science Foundation of China (Nos.91846205, 61772316, 61907026), Innovation Methods Work Special Project (No. 2020IM020100), Science and Technology Development Plan Project of Shandong Province (No. 2019JZZY020505), Key Research & development Project of Shandong Province (No. 2019GGX101009), Shandong-Chongqing Technological Collaboration Plan (No. cstc2020jscx-lyjsAX0010), and Project of Shandong Province Higher Educational Science and Technology Program (No. J18KA392).

## Conflict of Interest

The authors declare that the research was conducted in the absence of any commercial or financial relationships that could be construed as a potential conflict of interest.

## Publisher's Note

All claims expressed in this article are solely those of the authors and do not necessarily represent those of their affiliated organizations, or those of the publisher, the editors and the reviewers. Any product that may be evaluated in this article, or claim that may be made by its manufacturer, is not guaranteed or endorsed by the publisher.

## References

[B1] Barkhordari M. Niamanesh M. (2015). ScaDiPaSi: an effective scalable and distributable mapreduce-based method to find patient similarity on huge healthcare networks. Big Data Res. 2, 19–27. 10.1016/j.bdr.2015.02.004

[B2] Chan L. W.-C. Chan T.-K. Cheng L. Mak W. (2010). Machine learning of patient similarity: a case study on predicting survival in cancer patient after locoregional chemotherapy, in IEEE International Conference on Bioinformatics and Biomedicine Workshops (HongKong), 467–470. 10.1109/BIBMW.2010.5703846

[B3] Che C. Xiao C. Liang J. Jin B. Zho J. Wang F. (2017). An RNN architecture with dynamic temporal matching for personalized predictions of Parkinson's disease, in Proceedings of the 2017 SIAM International Conference on Data Mining (SDM) (Houston, TX), 198–206. 10.1137/1.9781611974973.23

[B4] Cheng R. Huang Z. Zheng Y. Yan J. Wong K. Y. Ng E. (2017). Meta structure: computing relevance in large heterogeneous information networks, in Asia-Pacific Web (APWeb) and Web-Age Information Management (WAIM) Joint Conference on Web and Big Data (Beijing), 3–7.

[B5] Cheng Y. Wang F. Zhang P. Hu J. (2016). Risk prediction with electronic healthrecords: a deep learning approach, in Proceedings of the 2016 SIAM International Conference on Data Mining (Miami, FL), 432–440. 10.1137/1.9781611974348.49

[B6] Gottlieb A. Stein G. Y. Ruppin E. Altman R. B. Sharan R. (2013). A method for inferring medical diagnoses from patient similarities. BMC Med. 11:194. 10.1186/1741-7015-11-194 24004670 PMC3844462

[B7] He J. Bailey J. Zhang R. (2014). Exploiting transitive similarity andtemporal dynamics for similarity search in heterogeneous information net-works, in International Conference on Database Systems for Advanced Applications (Bali), 141–155. 10.1007/978-3-319-05813-9_10

[B8] Hu L. Gong Y. Xing H. Wang F. (2019). Semantic representation with heterogeneous information network using matrix factorization for clustering in the internet of things. IEEE Access 7, 31233–31242. 10.1109/ACCESS.2019.2903310

[B9] Huai M. Miao C. Suo Q. Li Y. (2018). Uncorrelated patient similarity learning, in Proceedings of the 2018 SIAM International Conference on Data Mining (San Diego, CA), 270–278. 10.1137/1.9781611975321.31

[B10] Kasabov N. Hu Y. (2010). Integrated optimization method for personalised modelling and case studies for medical decision support. Int. J. Funct. Inform. Pers. Med. 3, 236–236. 10.1504/IJFIPM.2010.039123

[B11] Lee J. Maslove D. M. Dubin J. A. (2015). Personalized mortality prediction driven by electronic medical data and a patient similarity metric. PLoS ONE 10:e0127428. 10.1371/journal.pone.0127428 25978419 PMC4433333

[B12] Li C. Sun J. Xiong Y. Zheng G. (2014). An efficient drug-target interaction mining algorithm in heterogeneous biological networks, in Pacific-Asia Conference on Knowledge Discovery and Data Mining (Tainan; Taiwan), 65–76. 10.1007/978-3-319-13186-3_7

[B13] Li L. Cheng W.-Y. Glicksberg B. S. Gottesman O. Tamler R. Chen R.. (2015). Identification of type 2 diabetes subgroups through topological analysis of patient similarity. Sci. Transl. Med. 7:311ra174. 10.1126/scitranslmed.aaa9364 26511511 PMC4780757

[B14] Meng X. Shi C. Li Y. Zhang L. Wu B. (2014). Relevance measure in large scale heteroge-neous networks, in Asia-Pacific Web Conference (Changsha), 636–643. 10.1007/978-3-319-11116-2_61

[B15] Ng K. Sun J. Hu J. Wang F. (2015). Personalized predictive modeling and risk factor identification using patient similarity. AMIA Summits Transl. Sci. Proc. 2015, 132–136. 26306255 PMC4525240

[B16] Sarwar B. Karypis G. Konstan J. Riedl J. (2001). Item-based collaborative filtering recommendation algorithms, in Proceedings of the 10th international conference on World Wide Web (Hong Kong), 285–295. 10.1145/371920.372071

[B17] Sha Y. Venugopalan J. Wang M. D. (2016). A novel temporal similarity measure for patients based on irregularly measured data in electronic health records, in Proceedings of the 7th ACM International Conference on Bioinformatics, Computational Biology, and Health Informatics (Seattle, WA), 337–344. 10.1145/2975167.2975202 PMC7310718 32577627

[B18] Sharafoddini A. Dubin J. A. Lee J. (2017). Patient similarity in prediction models based on health data: a scoping review. JMIR Med. Inform. 5:e7. 10.2196/medinform.6730 28258046 PMC5357318

[B19] Shi C. Kong X. Huang Y. Yu P. S. Wu B. (2014). HeteSim: a general framework for relevancemeasure in heterogeneous networks. IEEE Trans. Knowledge Data Eng. 26, 2479–2492. 10.1109/TKDE.2013.2297920

[B20] Sun J. Wang F. Hu J. Edabollahi S. (2012). Supervised patient similarity measure of heterogeneous patient records. ACM SIGKDD Explorat. Newslett. 14, 16–24. 10.1145/2408736.2408740

[B21] Sun Y. Han J. (2010). Rankclus: integrating clustering with ranking for heterogeneous information network analysis, in Proceedings of the 12th International Conference on Extending Database Technology: Advances in Database Technology (Saint Petersburg), 565–576. 10.1145/1516360.1516426

[B22] Sun Y. Han J. (2011). Pathsim: meta path-based top-k similarity search in heterogeneous information networks, in Proceedings of the VLDB Endowment (Seattle, WA), 992–1003. 10.14778/3402707.3402736

[B23] Suo Q. Zhong W. Ma F. Ye Y. Huai M. Zhang A. (2018). Multi-task sparse metric learning for monitoring patient similarity progression, in 2018 IEEE International Conference on Data Mining (ICDM) (Singapore), 477–486. 10.1109/ICDM.2018.00063

[B24] Tsevas S. Iakovidis D. K. (2011). Fusion of multimodal temporal clinical data for the retrieval of similar patient cases, in 10th International Workshop on Biomedical Engineering (Kos), 1–4. 10.1109/IWBE.2011.6079049

[B25] Wang F. Hu J. Sun J. (2012a). Medical prognosis based on patient similarity and expert feedback, in Proceedings of the 21st International Conference on Pattern Recognition (Tsukuba), 1799–1802.

[B26] Wang F. Lee N. Hu J. Sun J. Ebadollahi S. (2012b). Towards heterogeneous temporal clinical event pattern discovery: a convolutional approach, in Proceedings of the 18th ACM SIGKDD International Conference on Knowledge Discovery and Data Mining (Beijing), 453–461. 10.1145/2339530.2339605

[B27] Wang F. Sun J. (2015). Psf: A unedified patient similarity evaluation framework through metric learning with weak supervision. IEEE J. Biomed. Health Inform. 19, 1053–1060. 10.1109/JBHI.2015.2425365 25910264

[B28] Wang R. Ma X. Jiang C. Ye Y. Zhang Y. (2020). Heterogeneous information network-based music recommendation system in mobile networks. Comput. Commun. 150, 429–437. 10.1016/j.comcom.2019.12.002

[B29] Xia E. Wang K. Zhang Y. Yu Y. Mei J. Li S. (2019). A data-driven clinical decision support system for acute coronary syndrome patient similarity, in 2019 IEEE International Conference on Healthcare Informatics (Xi'an), 1–6. 10.1109/ICHI.2019.8904614

[B30] Zhan M. Cao S. Qian B. Chang S. Wei J. (2016). Low-rank sparse feature selection for patient similarity learning, in 2016 IEEE 16th International Conference on Data Mining (ICDM) (Barcelona), 1335–1340. 10.1109/ICDM.2016.0182

[B31] Zhang C. Wang G. Yu B. Xie Y. Pan K. (2020). Proximity-aware heterogeneous information network embedding. Knowledge Based Syst. 193:105468. 10.1016/j.knosys.2019.105468

[B32] Zhang P. Wang F. Hu J. Sorrentino R. (2014). Towards personalized medicine: leveraging patient similarity and drug similarity analytics. AMIA. Jt Summits. Transl. Sci. Proc. 2014, 132–136. 25717413 PMC4333693

[B33] Zhu Z. Yin C. Qian B. Cheng Y. Wei J. Wang F. (2016). Measuring patient similarities via a deep architecture with medical concept embedding, in 2016 IEEE 16th Intermnational Conference on Data Mining (Barcelona), 749–758. 10.1109/ICDM.2016.0086

